# Spatiotemporal expression patterns of wheat amino acid transporters reveal their putative roles in nitrogen transport and responses to abiotic stress

**DOI:** 10.1038/s41598-017-04473-3

**Published:** 2017-07-14

**Authors:** Yongfang Wan, Robert King, Rowan A. C. Mitchell, Keywan Hassani-Pak, Malcolm J. Hawkesford

**Affiliations:** 1 Plant Sciences Department, Rothamsted Research, Harpenden, Herts AL5 2JQ UK; 2 Computational and Analytical Sciences Department, Rothamsted Research, Harpenden, Herts AL5 2JQ UK

## Abstract

Amino acid transporters have roles in amino acid uptake from soil, long-distance transport, remobilization from vegetative tissues and accumulation in grain. Critically, the majority of wheat grain nitrogen is derived from amino acids remobilized from vegetative organs. However, no systematic analysis of wheat AAT genes has been reported to date. Here, 283 full length wheat AAT genes representing 100 distinct groups of homeologs were identified and curated by selectively consolidating IWGSC CSSv2 and TGACv1 *Triticum aestivum* genome assemblies and reassembling or mapping of IWGSC CSS chromosome sorted reads to fill any gaps. Gene expression profiling was performed using public RNA-seq data from root, leaf, stem, spike, grain and grain cells (transfer cell (TC), aleurone cell (AL), and starchy endosperm (SE)). AATs highly expressed in roots are good candidates for amino acid uptake from soil whilst AATs highly expressed in senescing leaves and stems may be involved in translocation to grain. AATs in TC (TaAAP2 and TaAAP19) and SE (TaAAP13) may play important roles in determining grain protein content and grain yield. The expression levels of AAT homeologs showed unequal contributions in response to abiotic stresses and development, which may aid wheat adaptation to a wide range of environments.

## Introduction

Grain yield and protein content are dependent upon nitrogen accumulation in the grain. Greater than 70% of wheat grain nitrogen is remobilised and translocated from senescing leaves and stems^1, 2^, and amino acids represent the major transport form of organic nitrogen delivered to the endosperm cavity via the vascular strand^3, 4^. Large quantities of amino acids are imported into grain to meet the high nitrogen demand for synthesis of endosperm storage proteins, and for embryo development.

Plasma membrane transporters for amino acids are required for root uptake, xylem loading in the roots, phloem loading in leaves, and for nitrogen import into seeds. Amino acid transporters (AATs) have been identified in *Arabidopsis* ^5^, rice^6^, barley^7^, maize^8^, and other crops^9–11^. Based on sequence similarities and uptake properties, amino acid transporter families in plants comprise of two subfamilies: the amino acid/auxin permeases (AAAP) and the amino acid polyamine and choline transporters (APC)^12–15^. The AAAP subfamily can be further divided into general amino acid permeases (AAPs), lysine-histidine transporters (LHTs), proline transporters (ProTs), gamma-aminobutyric acid transporters (GATs), aromatic and neutral amino acid transporters (ANTs) and indole-3-acetic acid transporters (AUXs). A new group, amino acid transporter-like (ATL), was recently identified in rice^6^. The APC subfamily is grouped into three further subfamilies: cationic amino acid transporter (CATs), bidirectional acid transporters (BATs) and L-type amino acid transporters (LATs).

Within the amino acid transporter family, amino acid permeases (AAPs) have been functionally characterized in *Arabidopsis*. Eight members (AtAAP1–8) have been identified as amino acid permeases (AAP) that predominantly transport neutral and acidic amino acids with moderate affinity, with the exception of AtAAP3 and AtAAP5 which also transport basic amino acids^12, 16, 17^. AtAAP1 is highly expressed in the epidermal layer and embryo of seeds and regulates amino acid import to the embryo^18, 19^. AtAAP8 plays an important role in the uptake of amino acids into the endosperm during early seed development^20^. AtAAP3 and AtAAP5, which are expressed specifically in *Arabidopsis* root vascular tissue and in all root tissues, respectively, may be involved in amino acid uptake from soil^21, 22^. AtAAP2 and AtAAP6 have been suggested to function in xylem-phloem transfer^23, 24^.

Lysine-histidine transporters (LHTs) can transport lysine, histidine, neutral and acidic amino acids. Based on promoter-GUS localisation, LHTs have been suggested to be involved in import of amino acids into root and mesophyll cells (AtLHT1)^25^, as well as into pollen grains and other cells of reproductive floral tissue^26, 27^. AUXs/LAXs as major auxin influx carriers, regulate root gravitropism, root hair development (AUX1 and LAX1)^28, 29^, cotyledon vascular development, and leaf phyllotactixc patterning (LAX2)^30^. Proline-specific transporters (ProT) are widely expressed in *Arabidopsis*, but only AtProt2 is up-regulated by salt stress^31^. AtANT1, as an aromatic and neutral amino acid transporter, is expressed in all organs with the most abundance in flowers and in cauline leaves^32^.

In the APC subfamily, nine AtCATs have been identified in *Arabidopsis*. AtCAT5, as a high-affinity and basic amino acid transporter, may function in re-uptake of amino acids at the leaf margin, while AtCAT8 is preferentially expressed in young and rapidly dividing tissues, suggesting a role in the supply of precursors for other amino acids to the root and shoot meristems^33^. AtCAT1 is specifically localised in veins of leaf and root, and in developing siliques, indicating multiple roles in phloem physiology^34^. AtCAT2, which is localised in tonoplast, regulates soluble leaf amino acid concentrations^35^. AtBAT1, which is expressed in vascular tissues, may function in amino acid export from the phloem into sink tissues^36^. Five L-type amino acid transporters (LAT) have been identified^37^, and the localization and mutant analysis suggests that L-type amino acids play very important roles in mobilizing AAs from leaf mesophyll cells and green carpel cells under low C conditions (LAT4), and in amino acid homeostasis under abiotic stresses (LAT5)^38^.

Considerable progress has been made in identification and functional characterisation of amino acids transporters in plants, however little has been reported in wheat, one of the world’s most important cereal crops. The recent development of high throughput genomic and transcriptome sequencing technology has enabled the assembly of full length genes in wheat^39, 40^. In this study, 283 full length wheat AAT genes comprising 100 groups of homeologs (mostly triplets) are identified, and their spatio-temporal expression patterns in different organs, as well as their response to abiotic stress, are reported based on high-throughput transcriptome sequencing data. More importantly, AATs highly expressed in different grain cells (transfer cell, starch endosperm, and aleurone) are revealed, which provides preliminary molecular evidence for improved understanding of their functions in nitrogen transport pathways in wheat. There is a potential to improve nitrogen use efficiency and enhance wheat grain yields and protein contents by manipulating such amino acid transporters.

## Results

### Identification and full length assembly of AATs in wheat

In order to identify the amino acid transporters (AATs) presently found in IWGSC CSSv2 (Ensembl v30), the annotations were searched for amino acid transporter domains. A total of 324 AAT transcripts were identified initially (Supplementary Table S1). However, most of these were short partial sequences and subsequently multiple transcripts were found to represent one gene in later genomic sequence consolidation. To produce an improved phylogenetic tree, identify homeologous genes, and obtain accurate expression data, the full length AATs were assembled. A total of 283 wheat AAT genes representing 100 groups of homeologs were identified (Table 1 and Supplementary Table S2). All except 39 AAT genes were identified with at least one or more transcript IDs from IWGSC CSSv2. The 39 genes without corresponding transcript IDs which were identified, annotated and extracted from genomic sequence, were named as TaAAT (group name) with the sub-genome designation, such as TaAAP9.A. Homeologous genes were defined as those with nucleotide sequence similarities >90% from corresponding chromosomes in the different sub-genomes, and the majority of homeologous genes showed 95–98% similarity. In addition, most also had corresponding cM chromosome positions using the W7984 popseq data. In total, 89 out of 100 AAT homeologous groups comprised of three homeologs (triplets), and the remaining 11 AATs had one or two homeologs due to gene loss or lack of expression.

**Table 1:** Identification of amino acid transporters in wheat.

Subfamily	Group	AAT homeologous group No.	AAT gene No.
AAAP	AAP	22	60 (2, 6)*
	ATL	20	55 (2, 5)
	LHT	8	24 (0, 0)
	ANT	6	18 (0, 0)
	AUX	5	15 (0, 0)
	GAT	4	12 (1, 0)
	ProT	3	9 (0, 0)
	TTP	3	9 (0, 0)
APC	CAT	11	31 (0, 2)
	BAT	7	19 (2, 2)
	LAT	11	31 (2, 2)
Total		100	283

^*^(pseudogene, missing homeologs).

Within the AAAP subfamily, two groups: amino acid permease (AAPs) and amino acid transporter like (ATL) dominate the transporters, containing 22 (60) and 20 (55) homeologous groups (genes) respectively, in which both have 2 pseudogenes. In contrast, LHT, ANT, AUX, GAT, and Prot exhibit few group members, and only one pseudogene was identified in the GAT group. In the APC subfamily, 11, 11, and 7 homeologous groups from CAT, LAT and BAT including four pseudogenes respectively were identified (Table1).

### The distribution of AATs on chromosomes

**Figure 1:**
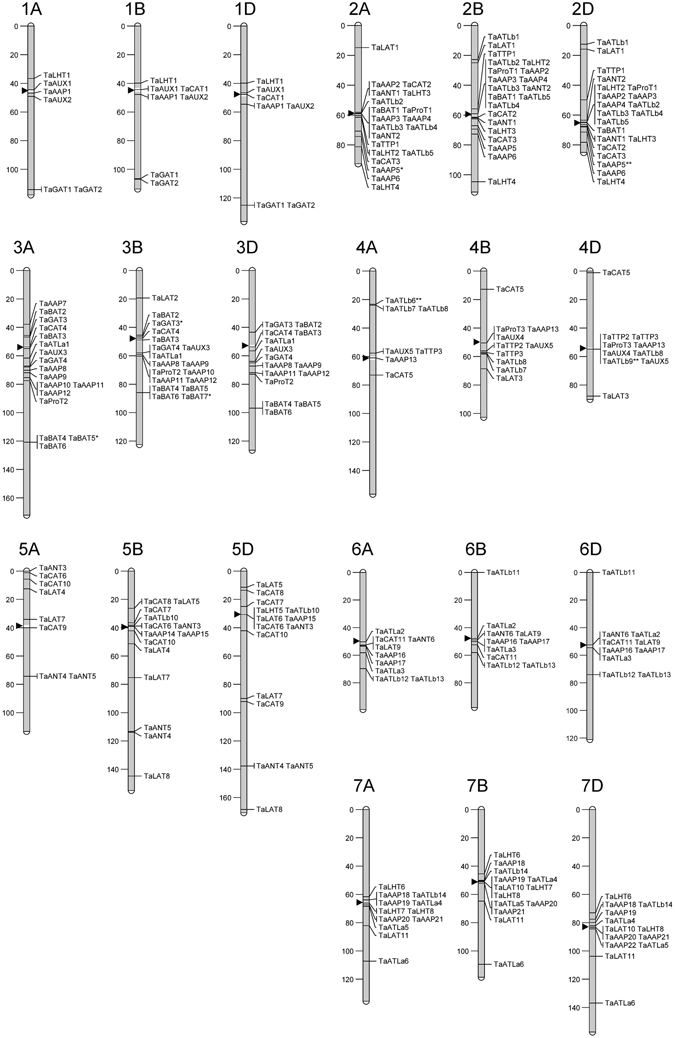
The distribution of AATs on wheat chromosomes using the genetic mapping distance, cM. The centromeres are indicated by arrowheads. The chromosomal positions used to generate the image are shown in Supplementary Table S5.

To determine the distribution of AATs on chromosomes, total of 263 genes were mapped on the wheat chromosomes (Fig. 1). Most of AAT genes are distributed on chromosomes 2 (62 genes), 3 (50 genes), and 7 (38 genes). By contrast, chromosome 1 has the least genes (20 genes). The majority of AATs are located near to the middle of the chromosomes, close to the centromere, and few genes are present at the ends of chromosomes.

### Phylogenetic analysis of amino acid transporters

**Figure 2:**
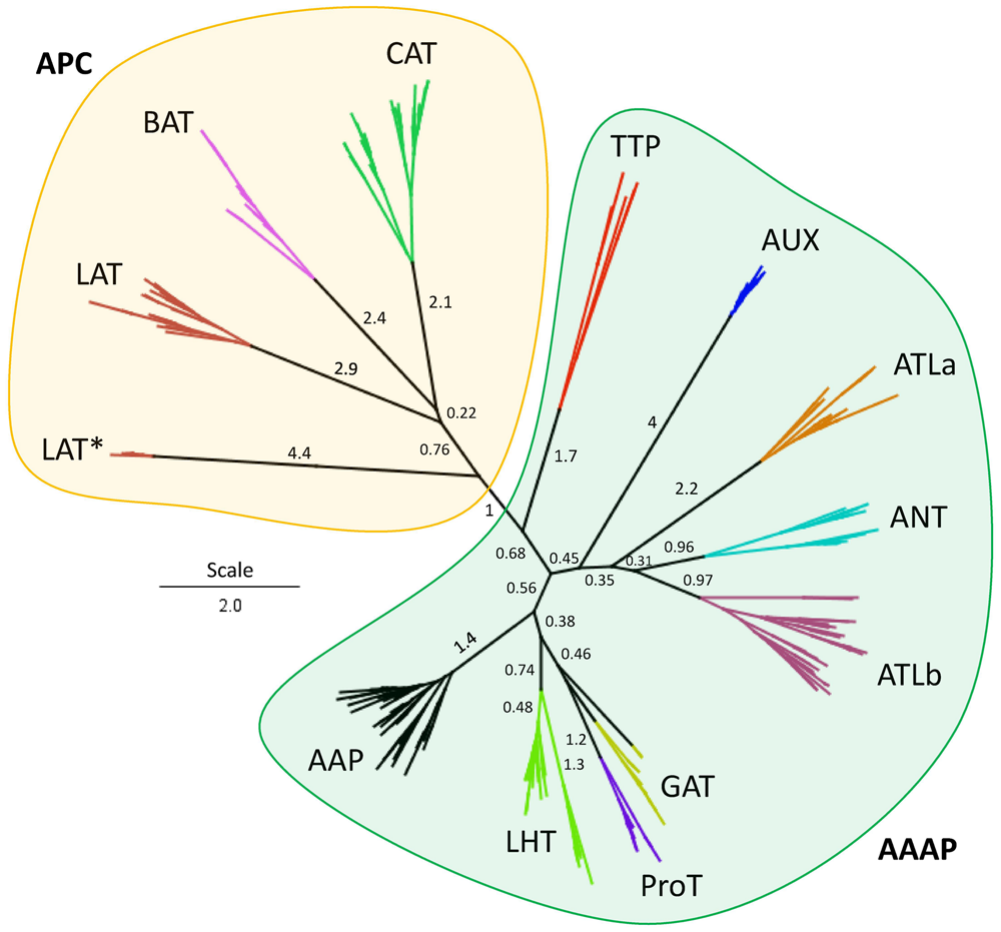
Phylogenetic tree of AAPs. 98 single functional genes of wheat AATs (one from each homeologous group) were aligned with rice and *Arabidopsis* using protein sequences. This tree was constructed using CLUSTALW and PHYML programs in Geneious. Detailed clustering was shown in Supplementary Fig. S1.

In order to evaluate the evolutionary relationship of wheat AATs to their orthologues in *Arabidopsis* and rice, 98 functional single homeologs from each homeologous group were aligned and a phylogenetic tree constructed using the neighbor-joining method. Two distinct clades representing two subfamilies (AAAP and APC) were observed (Fig. 2). The AAAP subfamily further divides into AAP, AUX, ANT, LHT, GAT ATL, and ProT groups, and the TTP group, not classified in rice^6^, is much closer to the AAAP subfamily and therefore was included in this group. The APC subfamily divides into three groups: CAT, LAT, and BAT. However, the LAT group splits into two branches due to a subset with an additional SLC12A domain (IPR018491) of 500 amino acids at the C-terminal end of the LAT* (TaLAT1,7,10) branch. No *Arabidopsis* orthologs in LAT* were found, suggesting that the expansion caused by the duplication occurred after separation of monocots and dicots. All the wheat AAT genes are closely clustered together with their orthologues from *Arabidopsis* and rice (Supplementary Fig. S1) in each group, which further confirmed their assignment, but also indicated they are very conserved.

### Gene expression profiling of AATs in wheat different organs

**Figure 3:**
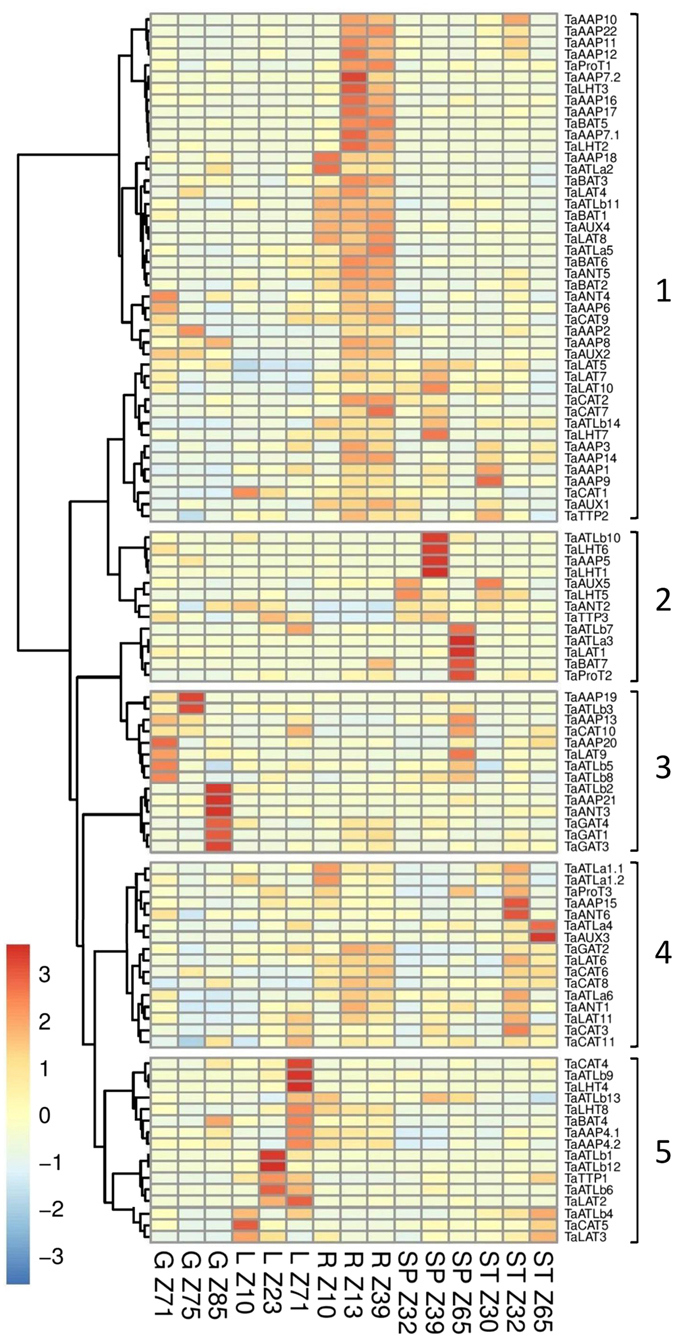
Spatiotemporal expression patterns of AAPs in different wheat organs scaled by row for Grain (G), Leaf (L), Root (R), Spike (SP), Stem (ST). The developmental stages are shown as Zadoks: Z10 (seedling stage), Z13 (three leaf stage), Z23 (tillering stage), Z30 (1 cm spike), Z32 (two nodes detectable), Z39 (flag leaf stage), Z65 (anthesis), Z71 (2 DPA, days post anthesis), Z75 (14 DPA), Z85 (30 DPA).

To determine the relative expression patterns of the identified AATs in the different wheat organs and at specific developmental stages, the RNA-seq datasets derived from root, stem, leaf, spike, and grains of the wheat cultivar, Chinese Spring^40^ were explored (Supplementary Table S3). Five clusters of highly expressed genes across organs and developmental stages were observed based on the heatmap (Fig. 3). 44 AATs (including 2 isoforms) in cluster 1 were preferentially expressed in root with TaAAP18 and TaATLa2 particularly abundant in very young roots (Z10, seedling stage), and TaLHT2, TaLHT3, TaProT1, TaATLa5, TaATLb11, TaAUX1, TaAUX4, highly expressed in mature roots (Z13, three leaves stage, and Z39 flag leaf stage). Cluster 2 had high expression mainly in spikes. AATs (TaATLa4, TaCAT5, TaATLb1,12) in cluster 5 are highly expressed in young leaves where nitrogen transport for seedling growth is important, while the AATs, TaATLb9, TaCAT4, TaLHT4, TaLHT8 and TaAAP4, have the highest expression at Z71 (2 days after anthesis), coincident with when the remobilized amino acids from senescing leaves are translocated to the grain. AATs in cluster 4 showed high expression in stem and also in root tissues, with TaAUX3 and TaATLa4 being abundant in stems at the Z65 (anthesis) development stage. AATs from cluster 3 and some from cluster 1 are preferentially and highly expressed in grains. TaAAP2, TaAAP19, TaAAP13 and TaATLb3 show high expression at 14 DPA and may be responsible for amino acid supply for protein synthesis at the middle grain filling stage, while TaAAP21, TaANT3, TaATLb2, and TaGAT3 with high expression at 30 DPA may play important roles in providing nitrogen during the late grain filling stage.

### Gene expression of AATs in different cell types of wheat grains

**Figure 4:**
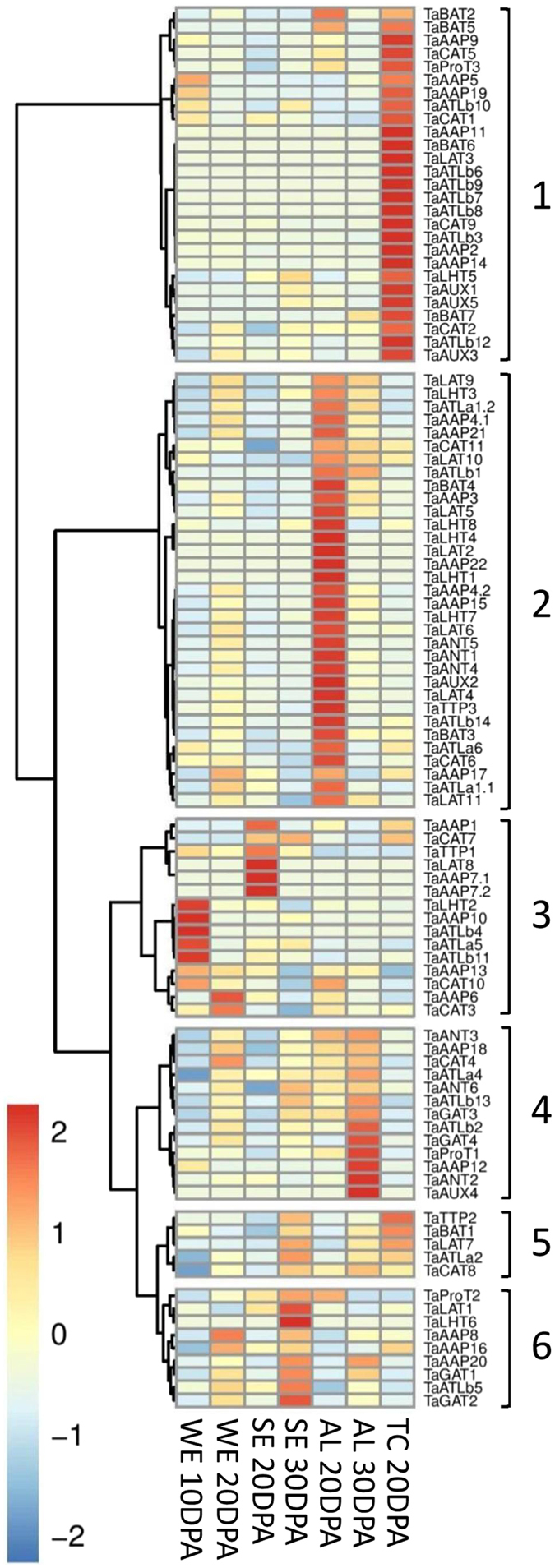
Spatiotemporal expression patterns of AAPs in different grain cells scaled by row in endosperm cells: transfer cell (TC), starchy endosperm (SE), aleurone cell (AL) at 20 and 30 DPA, and whole endosperm (WE) (including TC, SE, and AL) at 10 and 20 DPA. The TC and AL cells might be contaminated by SE tissue and result in high expression for AATs highly expressed in SE.

Wheat endosperm comprises three major types of cells: transfer cells (TC), aleurone cells (AL) and pure starchy endosperm (SE). In order to detect in which grain cells the AATs identified in whole grains are preferentially expressed, RNA-seq data derived from whole endosperm (TC, SE, and AL) at 10 and 20 DPA, TC at 20 DPA, AL at 20 and 30 DPA, and SE at 20 and 30 DPA^41^ was investigated (Fig. 4). Out of 27 TC-specific AATs, TaAAP2 and TaAAP19 showed extremely high expression in TC at 20 DPA (229 and 87 FPKM respectively in Supplementary Table S3), and reached peak expression at 20 DPA and 10 DPA in whole endosperm, suggesting a role in nitrogen uptake from the endosperm cavity by the TCs.

TaLAT5, TaAAP4, TaAUX2, TaAAP21, and TaANT1 with the highest expression in AL at 20 DPA in whole endosperm, may play roles in nitrogen supply for grain during the middle grain filling period. TaAAP13, and TaATLa5 were highly expressed in pure starchy endosperm (SE), indicative of a role in providing amino acids for storage protein synthesis. Due to the unpreventable contamination of AL and TC samples with SE during tissue dissection, those AATs with high expression in SE, may have an erroneous apparent high expression in TC or AT due to this contamination.

### Response of AATs to salt, heat, and drought stresses

**Figure 5:**
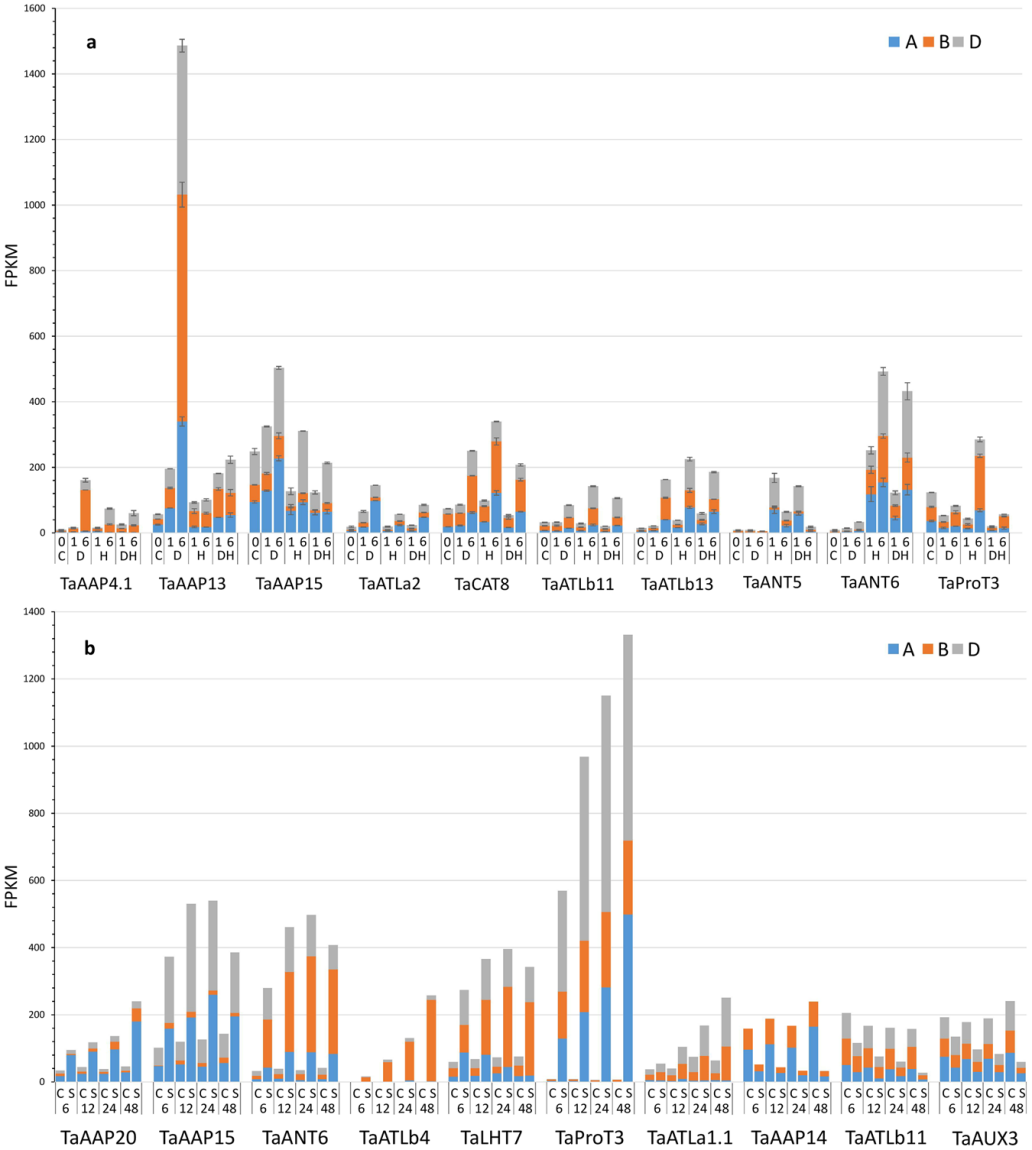
FPKM values of AATs and their homeologs highly induced by heat (H), drought (D), and combined heat + drought (DH) stress in 7-day-old leaf at 0 (control), 1, and 6 hours (5a), salt stress in 10-day-old root at 6, 12, 24, and 48 hours (5b). A, B, and D represent A, B, and D homeologs expression data.

To determine the response of amino acid transporters to abiotic stress, the expression levels of AAT genes were examined from 7 day old leaves under heat, drought, and heat and drought combined stress^42^, and for 10 day old roots under salt stress^43^. The AAT genes highly regulated by drought or heat are shown in Fig. 5a,b. Expression of TaAAP13 was induced to a very high level (26 fold at 48 h treatment) by drought stress in leaves (Supplementary Table S3). TaANT6 was up-regulated by both heat (57 fold at 6 h treatment) in leaves, and salt stress (15 fold at 24 h treatment) in roots. TaAAP14 and TaAUX3 were down-regulated by salt stress (7 and 4 fold respectively) in roots. There are three AATs (TaProt1–3) identified in the proline transport group, which responded differentially to stress. TaProt3, was down-regulated by drought (2 fold at 1 h treatment) in leaves, but showed extremely high expression (207 fold at 6 h treatment) under salt stress in roots. The expression of TaProt1 decreased three times under salt stress compared with normal condition in roots. In contrast, TaProT2 expression levels remained very low in leaves and roots and was also down-regulated under stress conditions.

### The expression of homeologs during development, and responses to abiotic stress

The hexaploid nature of modern wheat results in the existence of three homeologs for most wheat genes which may be expected to show similar expression patterns during development under different environments. In this study, unequal contributions of the three homeologs to expression from AATs were observed in response to abiotic stress and development (Fig. 5a,b and Supplementary Fig. S2). Homeologs from the B genome of TaAAP4.1, TaLHT7, TaATLb4 and TaAAP13 were more responsive to drought or salt stress than those from A and D genomes. For example, A, B, and D genomes of TaAAP4.1 contributed to 32%, 40% and 28% of expression in leaves under normal conditions, but accounted for 3.1%, 78.5% and 18.4% of expression under 6 h drought stress in leaves (Supplementary Table S3). In contrast, A and D homeologs of TaAAP15 dominated gene expression both in development and in response to salt, heat or drought stresses. In TaANT6, the B genome homeolog contributed the greater proportional expression response to 48 h salt stress (61.9%), but the D genome homeolog (46.8%) was more responsive than the B genome homeolog (22.7%) under 6 h drought and heat stress, indicating that individually the three homeologs might play different roles in adaption to environments.

Differing expression patterns of three homeologs were also observed in organs and grain cells (Supplementary Table S3 and Fig. S2). For example, the D homeologs of TaLHT8 and TaBAT4 showed high expression in leaf at Z71 (Supplementary Fig. S2c), and the A homeologs of TaLAT6 and TaAAP21 were highly expressed in aleurone cells at 20 DPA (Supplementary Fig. S2d).

### Confirmation of selected AATs expression

**Figure 6:**
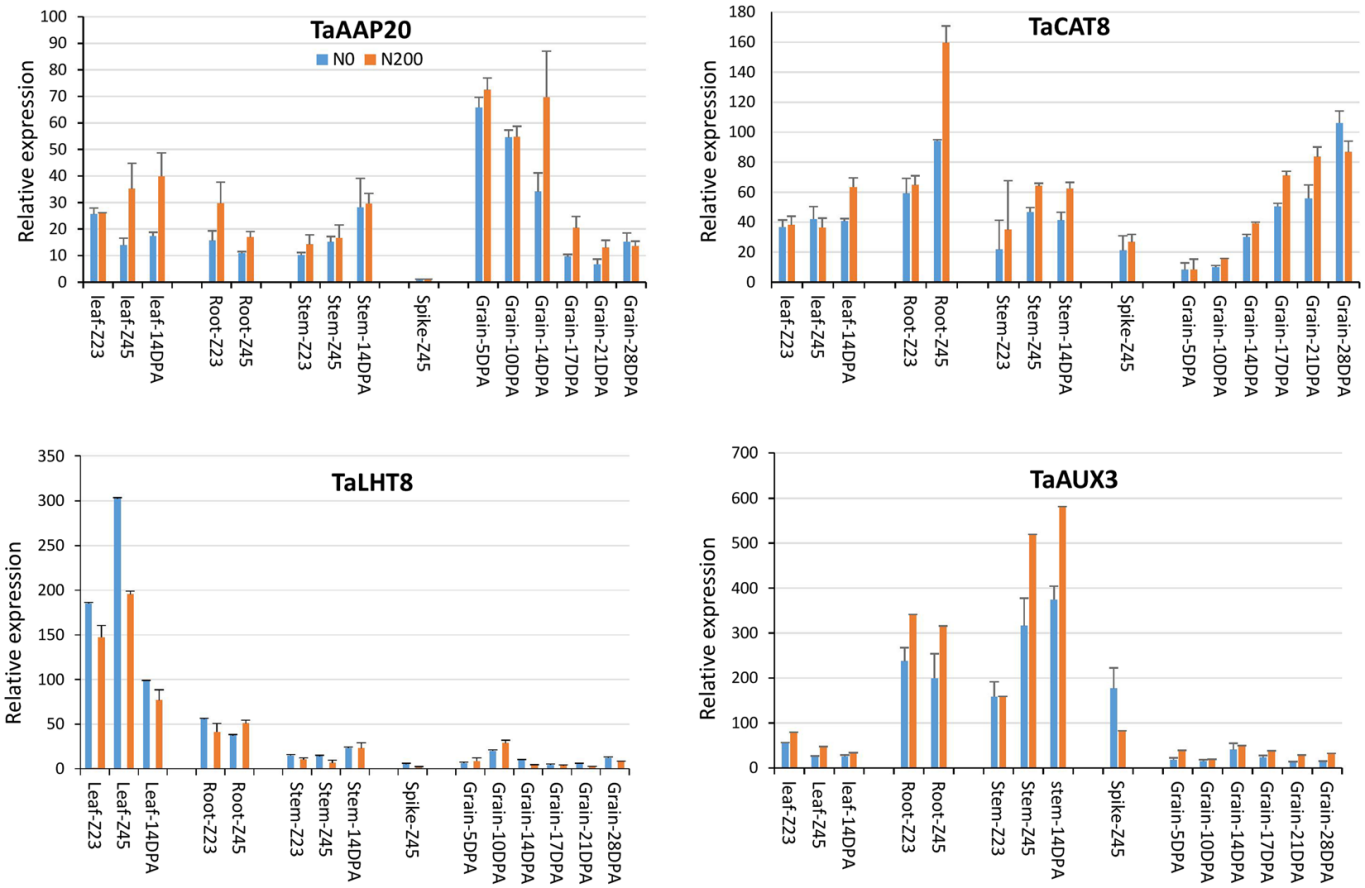
The four AATs representing high expression levels in different organs were confirmed by qPCR under two nitrogen applications at N0 (no nitrogen) and N200 (200 kg/ha nitrogen). Error bars represent standard errors.

To support the AAT expression patterns determined by RNA-seq data, four AATs representing contrasting expression patterns in different organs were selected for qPCR analysis for confirmation of expression. Primers were non-homeolog specific so values represent the sum of the homeologs expression for each AAT. RNA samples were isolated from root, leaf, stem, spike and whole grains of the bread wheat cultivar Hereward under two nitrogen applications and used for qPCR assays. The expression patterns of the four genes in different organs (Fig. 6) are consistent with the RNA-seq FPKM expression patterns (Supplementary Fig. S3). The transcripts of TaAAP20 were abundant before middle grain filling stage, and levels of TaCAT8 were highest at the late grain filling stage. TaLHT8 and TaAUX3 were highly expressed under the two nitrogen levels in vegetative organs leaves and stems, respectively.

## Discussion

64 and 85 amino acid transporters have been reported in *Arabidopsis* and rice, respectively^6, 44^. In this study, 283 full length AAT genes (100 homeologous groups) were identified and assembled from wheat genome data using bioinformatics methods. These AATs showed a favoured distribution on chromosomes 2, 3 and 7, and were centrally located close to the centromeres. While polyploidy is one mechanism by which gene family copy numbers expand, tandem duplication often resulting from unequal crossing-over is the most commonly evaluated mechanism for gene family expansion^45^. In 85 rice AATs, 55% genes came from segmental and tandem duplications, which contributed almost equally to the expansion of the OsAAT gene family^6^. The larger numbers of AATs identified in wheat compared to *Arabidopsis* and rice most likely resulted from the contributions of gene duplication and polyploidisation events. All wheat AATs closely clustered together with the *Arabidopsis* and rice orthologs, suggesting they were very conserved during evolution. However, a small group of LATs with the longest protein sequences were only identified in wheat and rice and did not have any orthologs in *Arabidopsis*, indicating that the expansion occurred after the separation of the monocot and dicot lineage.

This genome-wide survey on AATs revealed differential expression in specific organs and grain tissues, implying that individual AATs may play unique roles in nitrogen transport pathways. Plant roots are able to acquire amino acids from the soil for plant growth. AtAAP1, AtAAP5 and AtLHT1 in *Arabidopsis* have been reported to function in acquisition of the neutral and cationic amino acids from the soil respectively^26, 46^. TaATLa2, TaLHT2, TaLHT3, and TaAAP18 of wheat showed high expression in early seedling root, while TaATla5 was predominantly expressed in roots during flag leaf stages, suggesting that they might play important roles in root uptake of individual amino acids for wheat growth at different developmental stages.

About 70% of wheat grain nitrogen originates from senescing leaves and stems^1, 2^ and are transported through phloem transport^3, 4^. Therefore, phloem loading and delivery of amino acids to the seed sinks is crucial for grain productivity and quality. AtAAP2 was localised in the phloem throughout the plant, and AtAAP2 T-DNA insertion lines showed a decrease in the amount of seed total nitrogen and seed storage proteins, demonstrating AtAAP2 functions in phloem loading and amino acid distribution to the embryo^23^. Antisense inhibition of StAAP1 expression in potato leaves also reduced amino acid content in potato tubers^47^. PtCAT11 was highly and preferentially expressed in poplar phloem tissues and may be involved in the transport of glutamine from senescing leaves to sink tissues such as stems^48^. Here, we found that TaLHT8 and TaCAT4 were highly expressed in senescing leaves, while TaAUX3 and TaATLa4 were most abundant in stems (peduncles) during grain filling, which is consistent with the situation for rice AAT orthologs^6^. This suggested that the AATs which were abundant in senescing vegetative tissues during grain filling may be responsible for transport of remobilised amino acids from source tissues (mainly flag leaves and peduncles) to sink grains for protein synthesis after anthesis.

Nutrient solutes are delivered by the vascular strand and released by nucellar projection transfer cells into the endosperm cavity^49, 50^. Thereafter, they are taken up by TC and subsequently transported to the endosperm to be used for protein synthesis, both apoplastically and symplastically. Aleurone cells (AL) are enriched in micronutrients, lipids, and proteins, therefore, identification of AATs in TC, SE and AL would provide molecular evidence to target further improvement of grain yield and quality by manipulation of specific amino acid accumulation in endosperm. In this study, TC specific AATs reached peak expression in endosperm at 14–20 DPA, when the highest nitrogen uptake capability is expected, which is just prior to endosperm storage protein gene expression^51, 52^. TC-specific TaAAP19 as an ortholog of AtAAP8 which is required for amino acid uptake into endosperm during early seed development^20^, may also play an important role in TC amino acid uptake in wheat grain. TaAAP13, TaATLa5 and TaCAT8 were highly expressed in the endosperm and may contribute to amino acid transport in the endosperm for storage protein synthesis. In contrast, AATs in the AL started to show increasing expression levels from 10 DPA when the AL cells matured, and then remained high to the middle (20 DPA) or end of grain filling (30 DPA). This is similar to the micronutrient transporter expression pattern in AL in barley^53^. In *Arabidopsis*, AtAAP1 is highly expressed in epidermal cells (transfer cells) of the embryo, affecting seed protein content by importing amino acids into the embryo^18^. The wheat ortholog TaAAP21 is highly expressed in AL, indicating TaAAP21 may have the same function for the transportation of nitrogen into the embryo. OsAAP6 expressed in the endosperm of rice regulated the grain protein content and nutritional quality in rice by decreasing grain protein content in knock-out lines and increasing grain protein in overexpression lines^54^. Overexpression of broad bean (*Vicia faba*) VfAAP1 in pea and *Vicia narbonensis* seeds demonstrated that 10–25% of total grain nitrogen was enhanced by increasing amino acid uptake of seeds^55^. This implies that increase of amino acid transport in sink grain by manipulation of sink-located amino acid transporters may enhance grain yield productivity, and improve grain quality.

Drought and salinity are two major osmotic stresses that may dramatically reduce crop growth and productivity. Plants accumulate osmotically active compounds including proline to balance the increased osmotic potential under drought or salt stress conditions^56^. Overproduction of proline resulted in increased tolerance to osmotic stress in tobacco plants^57^. Altered amino acid compositions in the cells by adjusting nitrogen transport in response to environments subsequently resulted in changes in amino acid transporter expression levels. Proline transporter (ProT) induction under drought or salt stresses has been reported in *Arabidopsis* ^31^, rice^6^ and poplar^58^. In wheat, TaProT3 as an ortholog of AtProT2, was strongly induced by salt stress in roots, while TaProt1 and TaProT2 were down-regulated or unchanged under salt or heat and drought stresses. TaAAP13, which is most closely related to the root phloem specific AtAAP3, was substantially upregulated in leaves by drought. In contrast, TaAAP14, TaATLb11, and TaAUX3 were down-regulated by salt stress. These results suggested these AATs play important roles in response to the abiotic stresses by adjustment of transport of different amino acids.

As an allohexaploid, three homeologs from A, B, and D genomes were expected to exhibit similar expression patterns. However, expression partitioning of homeologs in development and response to abiotic stress in wheat was recently revealed by RNA-seq transcriptome profiling studies^42, 43, 59^. AATs in wheat also showed differential expression patterns among homeologs, either in development or response to salt, drought and heat stresses. One single or two homeologs were often predominantly expressed under normal and stress conditions. The uneven contribution of homeologs involved in mannitol synthesis has been reported in *Coffea arabica* ^60^ and the expression of the alcohol dehydrogenase A (AdhA) gene homeologs in allotetraploid cotton diverged significantly under multiple stresses^61^. The specific expression of homeologs during organ development and in response to stress conditions may benefit polyploid plants by allowing additional flexibility of response at the transcript level.

In this paper, a total of 283 complete, curated AATs wheat sequences have been obtained, and AATs highly expressed in specific organs and grain tissues were identified. Further research is required to investigate their cell localisation by RNA *in situ* hybridisation and promoter-GUS, and functional characterization by reverse genetic tools and substrate transport in yeast. To achieve both an improved grain yield and quality, increasing the source-sink nitrogen translocation via optimising phloem transport and amino acid accumulation in the grain would be promising approaches. TaAAP2, TaAAP13, and TaAAP21 were identified as the most promising candidate genes encoding AATs which might be involved in TC nitrogen uptake, endosperm N transport for protein synthesis, and AL nitrogen accumulation. Further investigations in our lab are ongoing to investigate their functions in the nitrogen transport pathway within the grain, for grain yield and for quality improvement. Overall this study provides novel information about AAT expression in wheat, and proposes hypotheses on their role in N transport. However, detailed experimentation is required to test these hypotheses and establish the biological functions of wheat AATs.

## Materials and Methods

### Identification of amino acid transporters

To identify the complete wheat amino acid transporter transcripts, extension of the genomic scaffolds was needed due to fragmentation of the genes over multiple contigs and missing sequences. The AA transporters annotated in the *Triticum aestivum* genome (IWGSC CSSv2 Ensembl v30) were searched using the Ensembl BioMart tool with InterPro ID’s: tryptophan/tyrosine permease (IPR018227), amino acid transporter transmembrane domain (IPR013057) and amino acid/polyamine transporter I (IPR002293). The corresponding 324 genomic sequences with flanking regions around the gene models of 5 kbp were extracted in fasta format. These genomic sequences were extended or gap filled by aligning the best hit blastn results from the TGACv1 (Ensembl v32) reference to the maximum length of those hits, within Geneious (v9.1.4)^62^. Redundant sequences, due to extensions of fragments were removed using 100% sequence identity via *de novo* assembly. Those sequences that showed partial alignment were inspected and removed if found to be due to containing unknown sequences (N’s). RNA-seq data^40^ from the main tissues were mapped to the resulting sequences using Tophat2 (v2.1.0) and transcripts assembled using cufflinks (v2.2.1). Cufflinks assembles the mapped RNA-seq reads into mRNA transcript annotations including the UTR but does not define the start and stop codons. These were manually curated in Geneious by taking the first ATG that had supporting RNA-seq evidence which resulted in the greatest length sequence, inclusive of the RNA-seq defined exon boundaries. During this process the exon boundaries were also confirmed and isoforms resolved where cufflinks had failed. The resulting transcripts were extracted and aligned using MAFFT^63^ (v7.017) with default settings within Geneious. Transcripts with greater than 90% sequence identity originating from homeologous chromosomes were defined as homeologs. Those below the threshold were inspected by examination of transcript and genomic alignments with homeolog sequences to identify causal reasons, which in each case were due to mutations such as SNPs or INDELs resulting in a pseudogene. Sequences lacking three homeologs or those containing coding gaps were completed by searching (blastn) the following databases in this order: (i) re-assembly of chromosome arm flow sorted IWGSC CSS reads which were assembled using SOAPdenovo2 (v2.04)^64^ with multi k-mer values from 61–81, (ii) TGACv1 genome assembly and (iii) W7984 genome assembly^65^. Scaffolds that contained small gaps had the IWGSC CSS reads mapped to the scaffolds and overlapping reads used to step-wise fill the gaps. A comparison of TGACv1 and IWGSC CSS v2, references in relation to the reassembled AAT scaffolds used in the consolidation process are shown in Supplementary Table S4. Three genes from IWGSC CSSv2 were excluded from the set because of the genomic scaffolds low length (<1.6 kbp) and consisting of a single exon sequence identical to other sequences on other chromosomes, so were suspected to be miss-assembly from contamination (Traes_5DS_0CBB9E7E6 (1,210 bp), Traes_2BL_AE31EE817 (1,585 bp), Traes_5BL_4A616899E (342 bp)). Four genes were excluded because of a lack of start/stop codon, a lack of supporting RNA-seq evidence, and lack of homeolog sequences; suggesting that these sequences are likely pseudogenes (TRAES3BF039300200CFD_g, Traes_6BS_714439F7F, Traes_2BL_A61DD929F, TRAES3BF068600070CFD_g). The nine pseudogenes that could be annotated consist of: 2 x INDEL deletion causing frameshift, 2 x INDEL deletion, 2 x INDEL insertion causing a frameshift, and 3 x SNP causing stop codon introductions (Supplementary Table S2). Resulting final genomic scaffolds, annotations and reassembled reference scaffolds that were used, are available in a data repository^66–68^.

### Phylogenetic tree

Alignments of one representative functional wheat homeolog protein (98) with the previously identified *Arabidopsis* and rice^6^ proteins were done using MUSCLE^69^ within Geneious, with default parameters: 8 iterations, pseudo tree rooting method, sequence weighting scheme was CLUSTALW^70^, clustering method was UPGMB. The first phylogenetic tree was constructed using PHYML^71^ with the options: substitution model as WAG, branch support as aLRTS, proportion of invariable sites set to estimated (not fixed), numbers of substitution rate categories as 4, gamma distribution as estimated (not fixed), optimized for topology, and topology search set to NNI. A second phylogenetic tree using 100 bootstraps, with a WAG substitution matrix, optimized for topology/length/rate, topology search NNI, numbers of substitution rate categories as 4, values from the first tree for gamma distribution matrix of 1.341 and proportion of invariable sites set to 0.

### Chromosomal localization of AATs

Corrected *T. aestivum* genomic scaffolds were queried using blastn to the W7984 reference and the POPSEQ cM positions were taken based upon best hit. There were 20 scaffolds without W7984 cM positions, which are not represented on the ideogram (Supplementary Table S5). Using the MapChart software^72^, the distribution of genes on chromosomes was drawn and modified with annotation. Those with stop codon SNPs were labelled with a “*” whilst those with INDELs were annotated with “**”.

### AAT expression analysis from RNA-seq data

Data was downloaded as is from ENA using the study accessions: SRP045409, ERP008767, SRP028357, SRP013449, ERP004505, SRP062745, and the urgi tissue data set from https://urgi.versailles.inra.fr/files/RNASeqWheat/ (Supplementary Table S6). Reads were mapped in Galaxy using bowtie2^73^ and default settings with the addition of the “-a” parameter to report all valid alignments. As reference, we used all CDS sequences from the *Triticum aestivum* TGACv1 (Ensembl version 31) and replaced the identified AATs with the curated AA transporter CDS sequences that we produced^74^. The mapped reads were converted to BAM format and sorted by read name (since Galaxy per default produced coordinated-sorted BAM) using SAMtools (v0.1.18)^75^. The FPKM’s were generated using Express^76^ (v1.5.1). Expression were averaged across replicates then summed across homeologs (Supplementary Table S3). The package pheatmaps (v1.0.8) and R (v3.1.2) were used to generate the heatmaps using FPKMs generated by Express with scaling by row to show peak tissue expression. The row clustering was performed using the ward.D method and Euclidean distance. Salt stress data was one replicate and drought/heat comprised two replicates.

### Harvest of materials and RNA extraction

The wheat variety Hereward was grown in the field trials at Rothamsted Research farm field in 2015, with either 200 kg/ha nitrogen or no nitrogen application. 50, 100, and 50 kg/ha nitrogen fertilizers (ammonium nitrate) were applied at tillering, stem extension, and flag leaf emergence stages respectively. Whole caryopses at 5, 10, 14, 17, 21, and 28 DPA (days post anthesis), roots and leaves as well as stems at Zadoks 23 (2–3 tillers stage), Zadoks 45 (booting stage), and 14 DPA were harvested and stored at −80 ^o^C for subsequent RNA extraction and real-time PCR.

Total RNA was isolated using the CTAB (cetyltrimethylammonium bromide) method as previously described^77^. Genomic DNA contamination was eliminated with RNase-free TURBO DNase (Ambion, Life Technologies). A 5 μg aliquot of total RNA was used for reverse transcription with SuperScript^TM^ III reverse transcriptase (Invitrogen) using anchored oligo (dT) 23 primers (Sigma-Aldrich).

### Real time PCR

Real time PCR was performed using a ABI7500 (Applied Biosystems) thermocycler. cDNA diluted 1:5 was used for RT–qPCR in a 25 μl reaction with SYBR^®^ Green JumpStart^™^ *Taq* ReadyMix (Sigma-Aldrich).

Ta54227 (Cell division control protein, AAA-superfamily ATPases) was used as an internal control gene as it showed the most stable expression across different wheat tissues and developmental stages^78^. The primers designed for qPCR are shown in Supplementary Table S7, and were used to amplify three homeologs. For each pair of primers, PCR efficiency was calculated in each run from a pool of all available cDNAs by using the LinRegPCR software^79^. All time points had three biological replicates. Normalised relative quantity (NRQ) of the target gene expression in relative to the internal control gene was calculated as following formula: NRQ = E.T^−Ct.T^/E.I^−Ct.I^, where E.T and Ct.T are the primer efficiency and Ct of target gene, and E.I and Ct.I are the primer efficiency and Ct of the internal control gene.

### Data Availability

All data generated or analysed during this study are included in this published article (and its Supplementary Information files).

## Electronic supplementary material

Supplementary Figures S1–S3

Supplementary Table S1

Supplementary Table S2

Supplementary Table S3

Supplementary Table S4

Supplementary Table S5

Supplementary Table S6

Supplementary Table S7
