# Supplementary material for: Spatiotemporal expression patterns of wheat amino acid transporters reveal their putative roles in nitrogen transport and responses to abiotic stress
Source: Sci Rep. 2017 Jul 14;7:5461. doi: 10.1038/s41598-017-04473-3 (PMC5511167; doi:10.1038/s41598-017-04473-3)

Spatiotemporal expression patterns of wheat amino acid transporters reveal their putative roles in nitrogen transport and responses to abiotic stress

Yongfang Wan[1], Robert King[2], Rowan A.C. Mitchell[1], Keywan Hassani-Pak[2], Malcolm J. Hawkesford[1]

1: Plant Sciences Department, Rothamsted Research, Harpenden, Herts, AL5 2JQ, UK.
2: Computational and Systems Biology Department, Rothamsted Research, Harpenden, Herts, AL5 2JQ, UK.

Supplementary Figure S1.
Clustering of 98 wheat, rice, and *Arabidopsis* AATs.

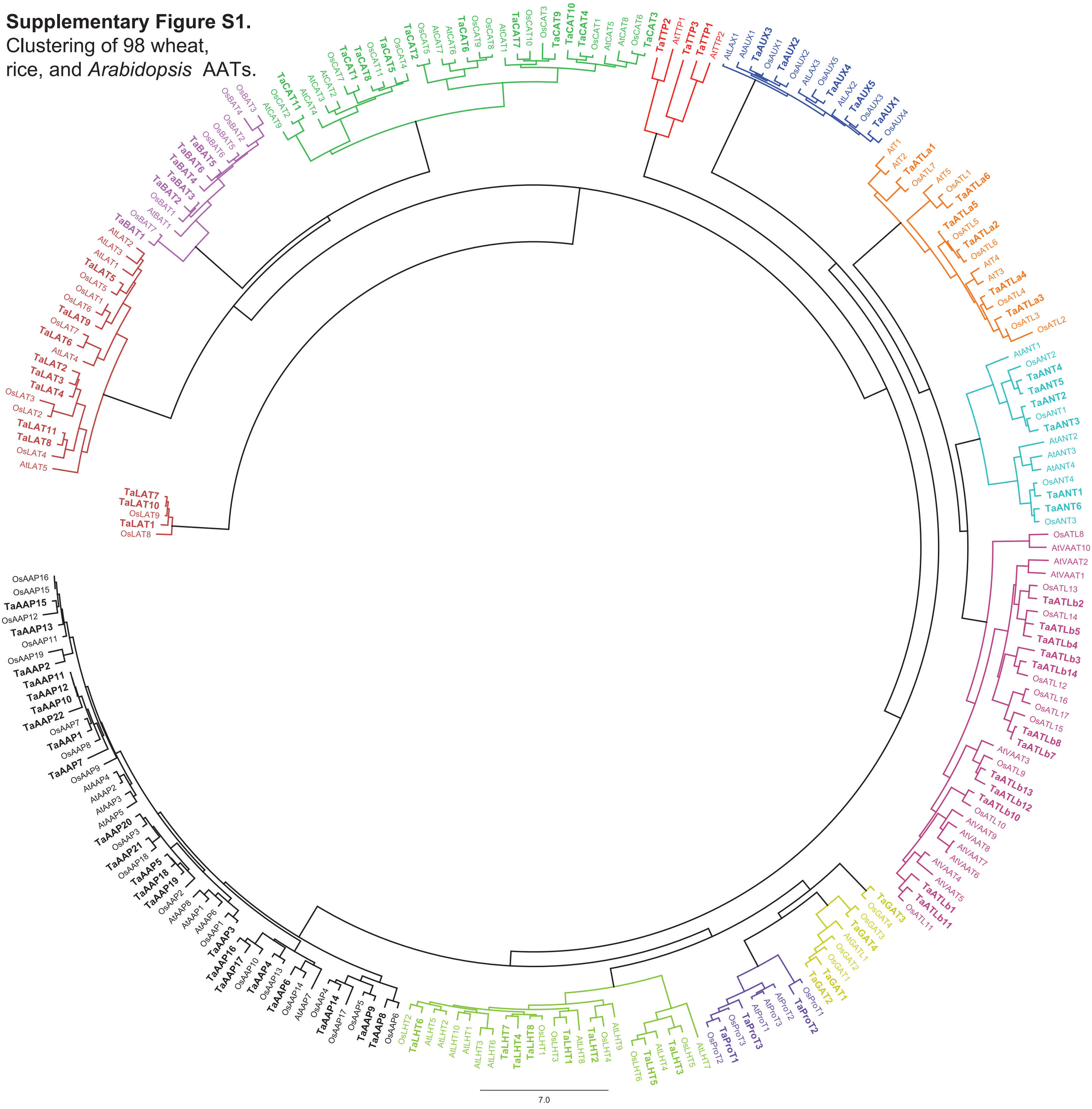

Supplementary Figure S2a.

Homeolog gene scaled (row) FPKM values from salt stress RNA−seq data.

Genes without at least a single column above FPKM 3.0 before scaling were not included.

Root tissue used.

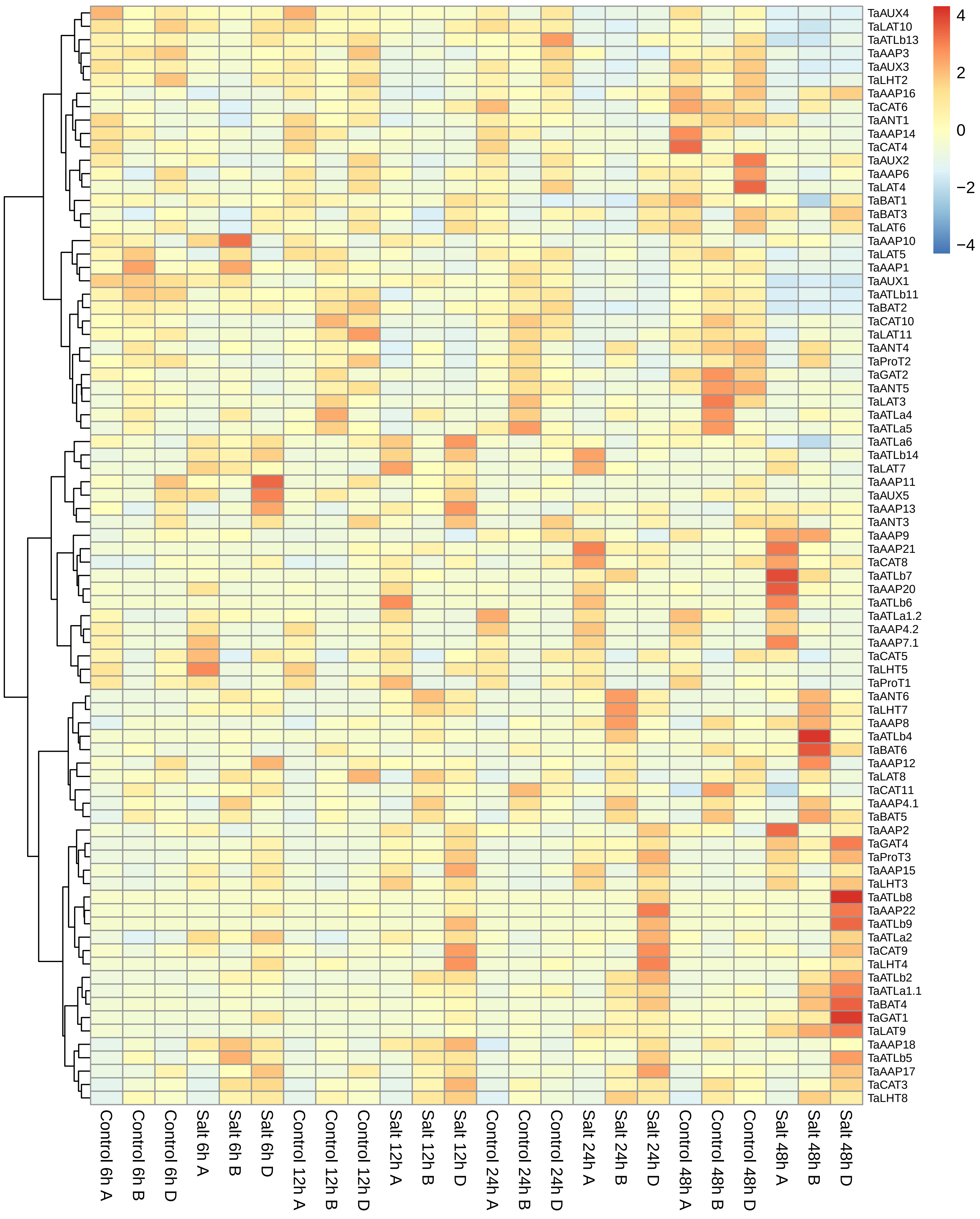

Supplementary Figure S2b.
Homeolog gene scaled (row) FPKM values from heat and drought RNA−seq data.
Genes without at least a single column above FPKM 3.0 before scaling were not included.
Leaf tissue used.

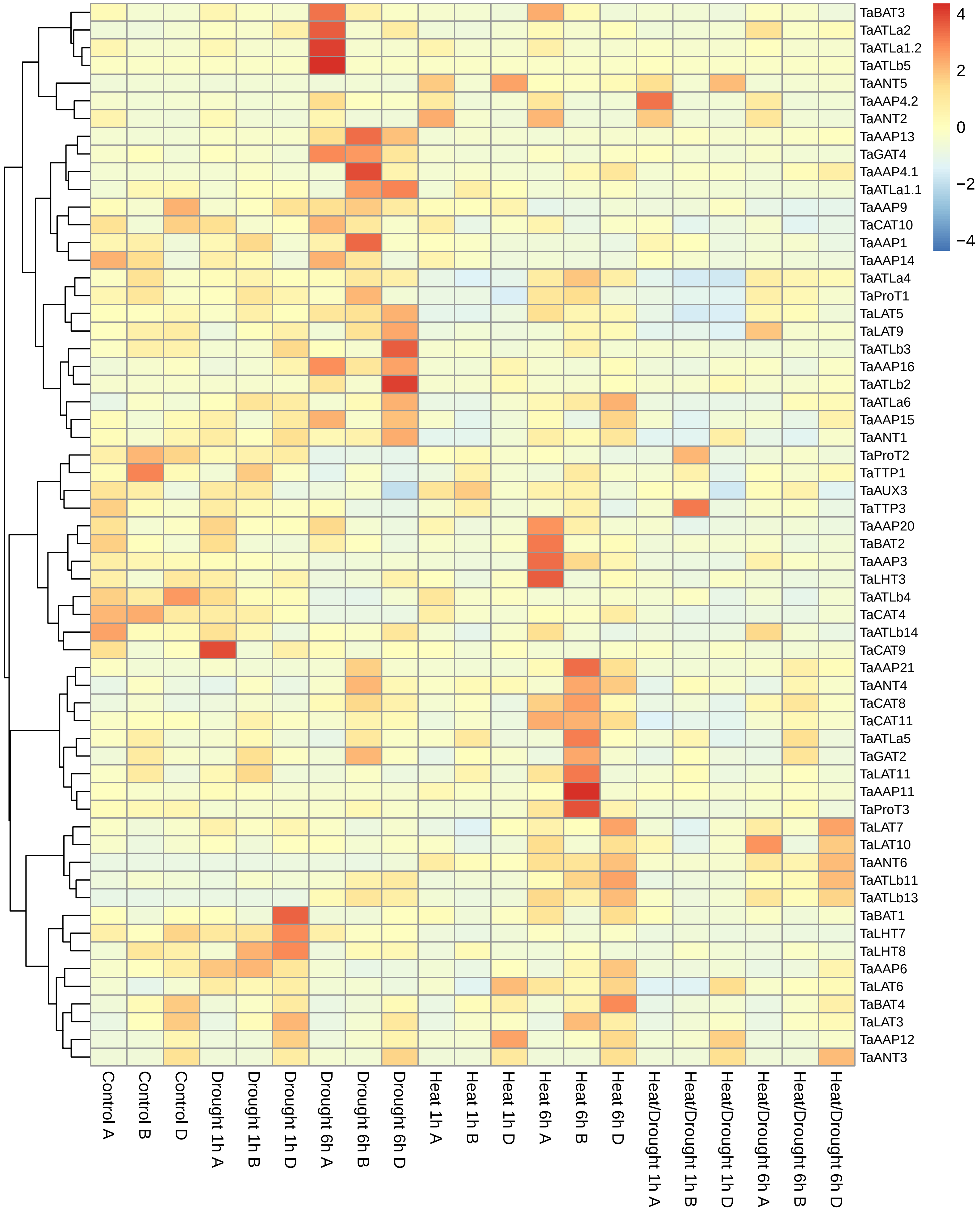

Supplementary Figure S2c.

Homeolog gene scaled (row) FPKM values from tissue specific RNA−seq data.

Genes without at least a single column above FPKM 3.0 before scaling were not included.

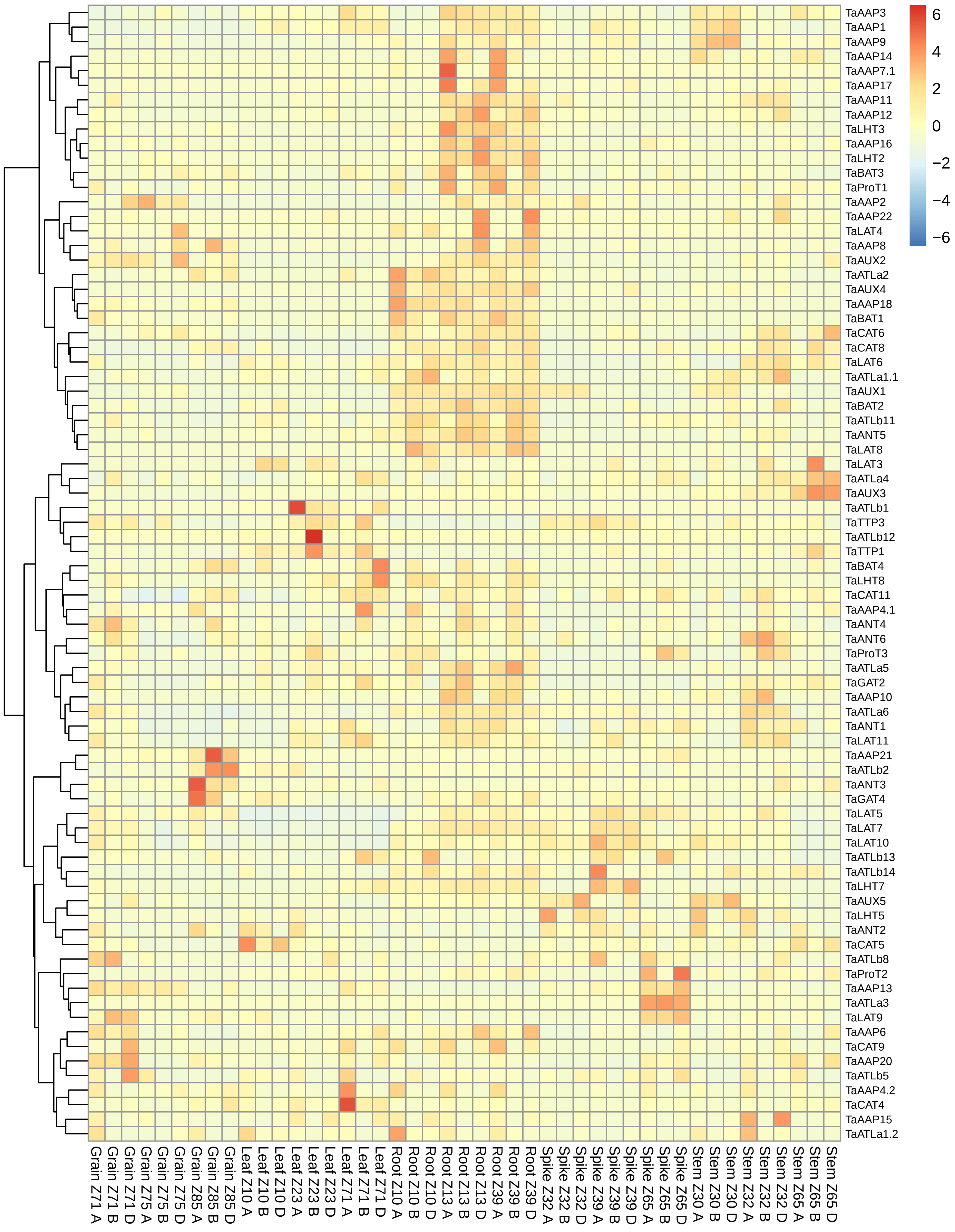

Supplementary Figure S2d.

Homeolog gene scaled (row) FPKM values from grain tissue specific RNA−seq data.

Genes without at least a single column above FPKM 3.0 before scaling were not included.

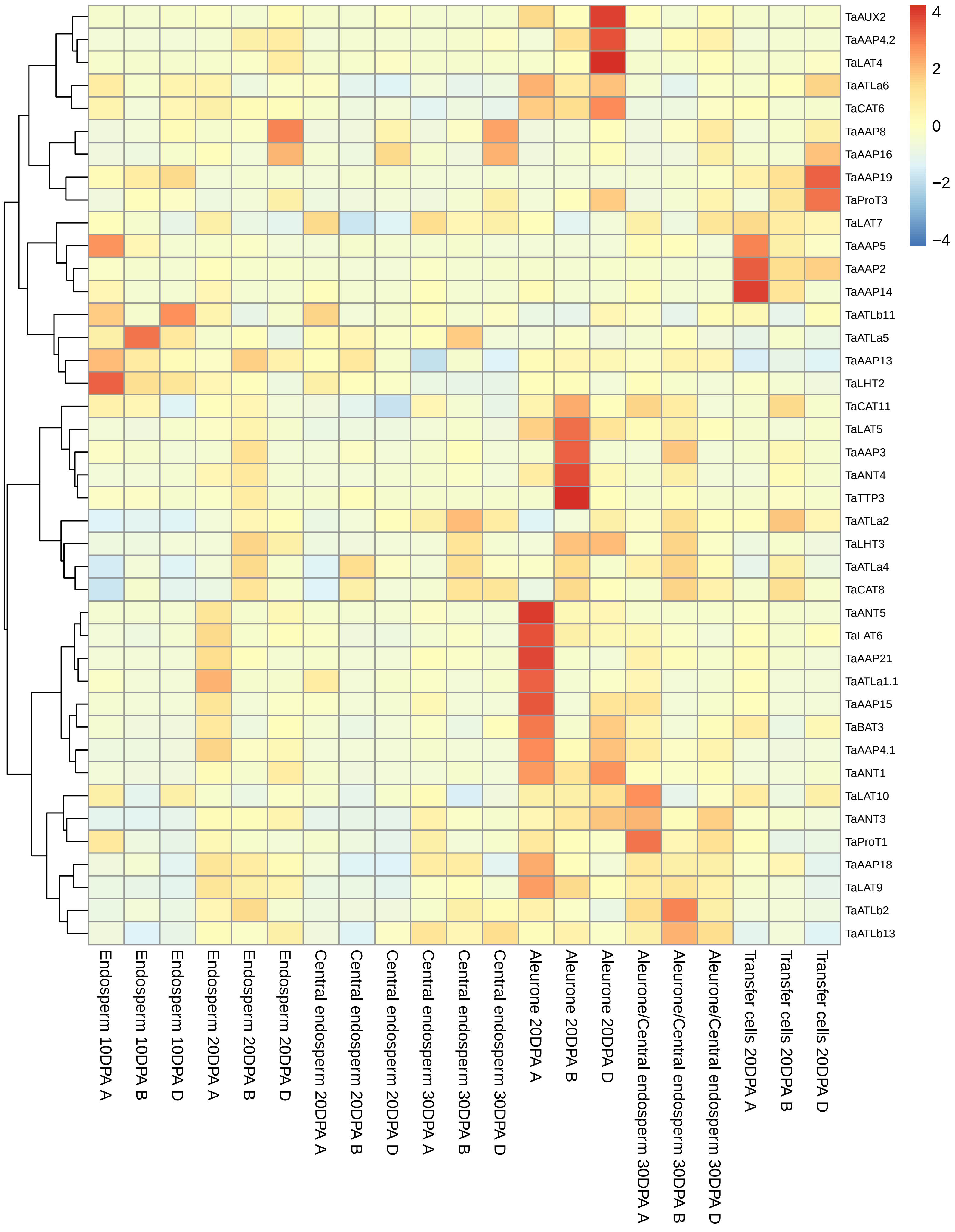

Supplementary Figure S3.

RNA-seq data expression (FPKM) from different organ tissues of four AATs that were used for qPCR in Figure 6.

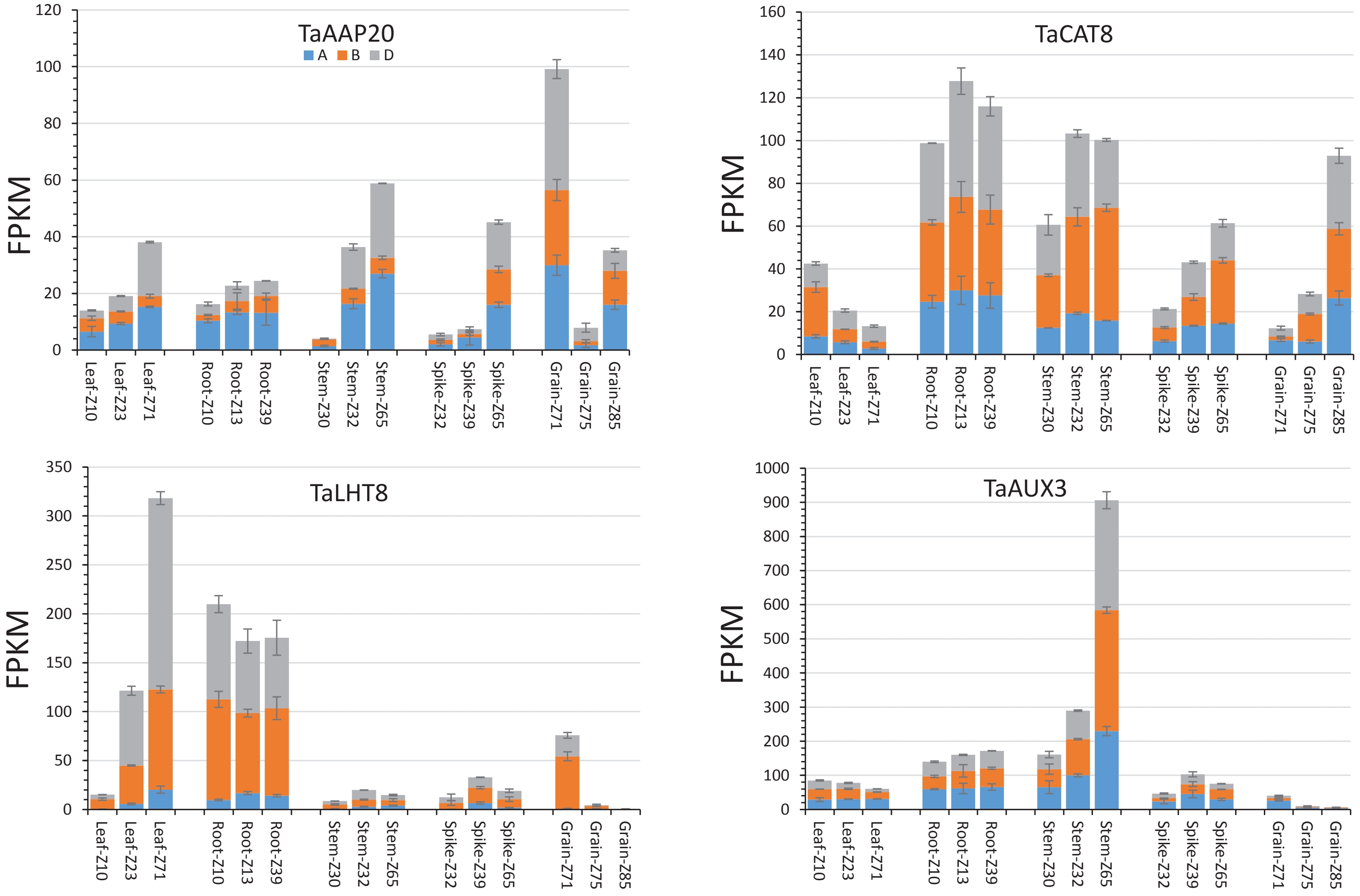

Supplement: Supplementary file 1 — Supplementary Figures S1–S3 [file 41598_2017_4473_MOESM1_ESM.pdf]
